# Physiologic and molecular consequences of endothelial Bmpr2 mutation

**DOI:** 10.1186/1465-9921-12-84

**Published:** 2011-06-22

**Authors:** Susan Majka, Moira Hagen, Thomas Blackwell, Julie Harral, Jennifer A Johnson, Robert Gendron, Helene Paradis, Daniel Crona, James E Loyd, Eva Nozik-Grayck, Kurt R Stenmark, James West

**Affiliations:** 1 Cardiovascular Pulmonary Research, University of Colorado Health Sciences Center, Denver, Colorado, USA; 2 Gates Center for Regenerative Medicine and Stem Cell Biology, University of Colorado Health Sciences Center, Denver, Colorado, USA; 3 Division of Basic Medical Sciences, Memorial University of Newfoundland, Newfoundland, Canada; 4 Division of Allergy, Pulmonary, and Critical Care Medicine, Vanderbilt University, Nashville, Tennessee, USA

**Keywords:** Pulmonary Arterial Hypertension, mouse model, pulmonary vascular disease

## Abstract

**Background:**

Pulmonary arterial hypertension (PAH) is thought to be driven by dysfunction of pulmonary vascular microendothelial cells (PMVEC). Most hereditary PAH is associated with BMPR2 mutations. However, the physiologic and molecular consequences of expression of BMPR2 mutations in PMVEC are unknown.

**Methods:**

In vivo experiments were performed on adult mice with conditional endothelial-specific expression of the truncation mutation Bmpr2^delx4+^, with age-matched transactivator-only mice as controls. Phenotype was assessed by RVSP, counts of muscularized vessels and proliferating cells, and staining for thromboses, inflammatory cells, and apoptotic cells. The effects of BMPR2 knockdown in PMVEC by siRNA on rates of apoptosis were assessed. Affymetrix expression arrays were performed on PMVEC isolated and cultured from triple transgenic mice carrying the immortomouse gene, a transactivator, and either control, Bmpr2^delx4+ ^or Bmpr2^R899X ^mutation.

**Results:**

Transgenic mice showed increased RVSP and corresponding muscularization of small vessels, with histologic alterations including thrombosis, increased inflammatory cells, increased proliferating cells, and a moderate increase in apoptotic cells. Expression arrays showed alterations in specific pathways consistent with the histologic changes. Bmpr2^delx4+ ^and Bmpr2^R899X ^mutations resulted in very similar alterations in proliferation, apoptosis, metabolism, and adhesion; Bmpr2^delx4+ ^cells showed upregulation of platelet adhesion genes and cytokines not seen in Bmpr2^R899X ^PMVEC. Bmpr2 mutation in PMVEC does not cause a loss of differentiation markers as was seen with Bmpr2 mutation in smooth muscle cells.

**Conclusions:**

Bmpr2 mutation in PMVEC *in vivo *may drive PAH through multiple, potentially independent, downstream mechanisms, including proliferation, apoptosis, inflammation, and thrombosis.

## Background

Pulmonary arterial hypertension (PAH) is a lethal disorder characterized by pulmonary vasoconstriction and vascular remodeling, leading to progressively worsening right ventricular strain, and eventual right heart failure[1]. Early molecular and physiologic events in the development of the idiopathic form of PAH (IPAH) are not known. However, in most theories of etiology, the pulmonary microvascular endothelial cell (PMVEC) plays a central role, either because of aberrant signaling to the smooth muscle[2], excess proliferation which fills in vessels[3], or excess apoptosis which causes vessels to drop out[4].

The familial form of PAH is usually caused by mutations in the type 2 receptor for the bone morphogenetic protein signaling pathway, BMPR2[5,6], and there is substantial evidence that suppression of the BMP pathway is involved in all idiopathic PAH[7]. However, detailed effects of BMPR2 mutation in endothelium, either in vivo or in vitro, are not known.

BMPR2 signals through multiple downstream pathways[8]. In canonical signaling, when a dimer of BMPR2 receptors forms a complex with a dimer of type 1 receptors in the presence of ligand, the type 2 receptors phosphorylate and thus activate the type I receptor, allowing the type 1 receptor to phosphorylate SMAD transcription factors 1, 5, or 8. The SMAD transcription factor complexes with SMAD4, and enters the nucleus to transcribe targets. However, BMPR2 also possesses a long cytoplasmic tail, well conserved by evolution but found in no other TGFb superfamily receptor. This tail domain is not required for SMAD signaling[9], and signals through other, currently poorly understood, pathways[10,11]. Mutations in human patients can fall anywhere in the gene[12]. Determining the common molecular consequences of SMAD dependent and cytoplasmic tail dependent signaling is thus important to determining how BMPR2 mutation predisposes to disease.

We have previously reported physiologic and molecular consequences in smooth muscle of inducible transgenic overexpression of Bmpr2 mutants with defects in SMAD-dependent (and cytoplasmic tail-dependent (Bmpr2^R899X^) signaling[9,13,14]. These transgenic mice rely on the fact that the Bmpr2 receptor works as a dimer; thus, overexpression of a mutant protein inhibits expression of the native protein in two ways - by acting as a decoy receptor, and by binding native receptor into a nonfunctional complex.

The goal of this study is to complete a first description of the physiologic and molecular consequences of the different classes of Bmpr2 mutation in endothelium. Yelle et al have already reported physiologic consequences of our Bmpr2^R899X ^mutant under an endothelial promoter[15]. In this report we thus express our doxyycline-inducible Bmpr2^delx4+ ^mutant in adult mice under the control of a previously published Tie2 promoter[16-18] and perform hemodynamic and histologic examination of consequences. To determine molecular consequences, we cultured PMVEC from control, Bmpr2^R899X^, and Bmpr2^delx4 ^mice, and performed expression analyses using Affymetrix arrays. We found that Tie2-rtTA x TetO_7_-Bmpr2^delx4 ^mice developed variably elevated RVSP, associated with increased muscularization particularly of small arteries, increased proliferation, infiltration of a variety of inflammatory cell types, increased thrombosis, and increased apoptosis. Expression analysis found that Bmpr2^delx4 ^and Bmpr2^R899X ^mutations caused very similar changes in proliferation, apoptosis, adhesion, and metabolism, but were quite different in their effect on inflammatory and thrombotic genes.

## Methods

### Generation of Tie2-rtTA x TetO_7_-Bmpr2^delx4+ ^mice

Both transgenes used for this double-transgenic mice have been independently previously published. The Tie2-rtTA mouse was originally generated for study of neovascular retinopathy, and was shown to be endothelial specific in that context[16-18]. The TetO_7_-Bmpr2^delx4+ ^mice were first described and validated crossed to a smooth muscle-specific SM22-rtTA second transgene[14]. Both mice were on an Fvb/n strain background. Double transgenic mice are doxycycline inducible; they express very little transgene until fed doxycycline, with expression levels in whole lung roughly equivalent to expression in the lungs of our previously published SM22-rtTA x TetO_7_-Bmpr2^delx4+ ^model[14].

### Mouse Phenotyping Protocol

Mice were given Avertin (500 mg/kg IP) to induce a surgical plane of anesthesia. The animals were then shaved to expose the surgical area. Mice were placed on a heated surgical table (Harvard Apparatus 872/1, 872/H, Holliston, MA) and secured with surgical tape. Systemic blood pressure and pulse was measured via a tail cuff and pulse transducer run through a PowerLab NIBP Controller (ADInstruments, Colorado Springs, CO). The surgical site was viewed using a Zeiss OpMi 1 surgical microscope. An incision of approximately 1 inch in length was made extending from the animal's chin down to the right armpit. The thyroid gland was then blunt dissected upwards to expose the underlying tissue and the right jugular vein. The jugular vein was then separated from surrounding tissue using dissecting forceps until the body of the vessel was completely free from adherent tissues. The cranial end of the jugular was tied off completely and a loose tie was then made at the caudal end of the exposed jugular using 4-0 braided silk suture. 4" Micro-dissecting scissors were then used to make a small incision in the medial aspect of the right jugular vein. A Millar 1.4 French pressure/volume micro-tip catheter transducer (SPR-839, Millar Instruments, Houston, TX) connected to a PowerLab/8S (ADInstruments) was then inserted through the incision and gently threaded down into the right ventricle. Proper placement within the ventricle was determined through observation of the pressure volume loop obtained from the catheter. The loose caudal suture was then tightened to secure the catheter in place. Once the catheter is properly placed, data was collected using Chart 5 for Windows (ADInstruments). Once blood pressure and volume data was collected, the caudal suture was re-loosened and the catheter removed for tissue inflation. 100U of heparin was then injected through the jugular incision to prevent clotting. The suture was then re-tightened to prevent bleeding. The animals were then removed from the surgical table to a dissecting area. All animal procedures were approved by the UCHSC animal care and use committee (IACUC).

### Mouse tissue collection and morphometry

After checking for insensitivity to toe-pinch, a paramedian sternotomy was performed, lungs were flushed with saline injected through the RV, the right lung was tied off, and the left lung was inflated using low-melt agarose injected through the trachea. The left lung was removed and fixed in 10% formalin and paraffin embedded. The right lung was flash-frozen in liquid nitrogen. Hearts were collected for weighing to determine right ventricular muscularization. Lung sections were stained for smooth muscle actin (SMA), counterstained with hematoxylin, and imaged using a Zeiss Axioscop 2 microscope. Twelve 20x microscopic fields were collected from each animal from which counts of fully or partially (less than 50%) muscularized vessels and vessel diameter were collected.

### Immunoflourescence and Carstair's Stain

Immunofluorescence was done as previously described using the following antibodies and dilutions. Monoclonal anti-alpha smooth muscle actin antibody was purchased from Sigma (St. Louis, MO) and used at a dilution of 1:1000. Rabbit polyclonal anti-smooth muscle actin was purchased from Abcam (Cambridge, MA) and used at a dilution of 1:250. Rabbit polyclonal anti CD45 was purchased from Santa Cruz Biotechnology (Santa Cruz, CA) and used at 1:100. Rabbit polyclonal CD3 epsilon was purchased from Abcam (Cambridge, MA) and used at a dilution of 1;200. Mac-3 antibody was purchased from BD Pharmingen (San Jose, CA) and used at a dilution of 1:200. Tissues processed for immunofluorescence were rinsed and placed in either Alexa Fluor 488 or 594 (Invitrogen, Carlsbad, CA) secondary antibody at a 1:1000 dilution for 1 hour at room temperature in the dark. Slides were then rinsed with PBS and coverslipped with Vectashield with DAPI (Vector Laboratories, Burlingame, CA). All staining was evaluated and digital images acquired using a Zeiss Axioskop 2 equipped with an AxioCam (Carl Zeiss Microscopy, Jena Germany). Carstairs' stain[19] for fibrin and platelets was done by IHC tech (LLC12635 Montview Blvd. Ste. 215 Aurora CO, 80045) Briefly, deparaffinized slides were incubated 5 minutes in ferric aluminum, rinsed, stained with Mayer's hematoxylin 5 minutes, rinsed, stained in picric acid- orange G solution for 30 minutes, rinsed, differentiated with 1% phosphotungstic acid, rinsed and stained with aniline blue solution for 1 hr and rinsed before mounting. This is a modification of picro-Mallory staining, resulting in orange to red fibrin, gray-blue platelets, bright blue collagen, red muscle, and red, green, or yellow red blood cells.

### Cell culture and siRNA

Murine pulmonary microvascular endothelial cells (MVECs) were isolated by collagenase digest of whole lung tissue and selected by cytometry using PECAM-1. The isolated cells were grown to ~60% confluency on 10 cm cell culture dishes in Endothelial Growth Media 2 (EGM2) containing 5% serum. Following VE-Cadherin and diLDL staining to confirm EC phenotype, transfections were done at passage 3 using 100 nM scrambled siRNA control (Qiagen, Valencia, CA) or siRNA to BMPR2 (Ambion). Lipofectamine and Plus reagents (Invitrogen, Carlsbad, CA) were mixed with Optimem media (Invitrogen) and siRNA as per manufacturer's instructions. EGM2 media was replaced with Optimem before lipid-RNA complexes were added to the cells and incubated for 2 hours at 37°C. A volume of EGM2 media containing 10% serum equal to that already on the plate was then added to the existing transfection media and the cells were incubated for and additional 24-48 hrs. Cells were subsequently harvested using the PARIS kit from Ambion (Austin, TX). To extract the BMPR2 from the membrane, SDS was added to a final concentration of 6%. Equal amounts of protein determined using BCA protein assay (Pierce, Rockford, IL) was loaded onto a 10% polyacrylamide gel with 10% glycerol and transferred to Hybond-P membrane (Amersham, Piscataway, NJ). BMPR2 is detected with a mouse monoclonal antibody diluted 1:150 (BD Transduction Laboratories, San Jose, CA) and visualized by chemiluminescence (ECL, Amersham, Piscataway, NJ).

### Apoptosis - Tissue

The fluorescein FragEL DNA fragmentation detection kit (Calbiochem, San Diego, CA) was used to look at fragmented DNA signifying apoptosis. The manufacturer's protocol was followed. Briefly, paraffin embedded lung tissue from Tie2- BMPR2^delx4+ ^and control mice were cut, put onto slides and deparaffinized as described above. Fragmented DNA was labeled using terminal deoxynucleotidyl transferase which binds to exposed 3'-hydroxyl ends left after fragmentation of DNA in response to apoptotic signals and catalyzes the addition of fluorescein-labeled and unlabeled deoxynucleotides allowing for visualization.

### Apoptosis - Cells

Plates (60 mm) were seeded with 3 × 10^5 ^mouse pulmonary microvascular endothelial cells in Minimum Essential-alpha (MEM-a) media (Invitrogen, Carlsbad, CA) supplemented with 20% FBS and incubated overnight. BMPR2 siRNA or scrambled siRNA (100 nM, Ambion) was transfected using Lipofectamine and Plus reagent (Invitrogen, Carlsbad, CA), cells were recovered with complete media at 2 h. At 72 h cells were trypsinized, washed with ice-cold PBS and resuspended in 1 ml of PBS. Apoptotic cells were labeled with YO-PRO-1, Vybrant Apoptosis Assay (Invitrogen, Carlsbad, CA) which stains apoptotic cells green fluorescent while necrotic cells stain red fluorescent with propidium iodide separating early apoptosis and necrosis using flow cytometry. To prevent apoptosis through the BMPR2 pathway, cells were seeded as described above. After overnight incubation, cells were washed with PBS and media replaced with MEM-a without FBS. At 24 h, 150 ng/ml BMP-2 (R&D systems, Minneapolis MN) was added and at 72 h cells were trypsinized, washed with ice-cold PBS and resuspended in 1 ml of PBS. Apoptotic cells were labeled with YO-PRO-1 (Vybrant Apoptosis Assay, Molecular Probes) and analyzed by flow cytometry.

### Generation of murine PMVEC

Immortomouse X Rosa26-rtTA2 X TetO_7_-Bmpr2^R899X ^or Immortomouse X Rosa26-rtTA2 X TetO_7_-Bmpr2^delx4+ ^triple transgenic mice (Immorto-Bmpr2^R899X^) were bred with transgenes verified by PCR genotyping of tail DNA. The immortomouse contains a transgenic insertion of the SV40 large T antigen, tsA58, under control of an interferon-inducible promoter[20]. When cells are grown at 33°C and interferon is added, the transgene is activated and the cells are immortalized and proliferate freely; at 37°C, this transgene is inert. The immortomouse therefore produces cells which proliferate as though they were immortalized at 33°C, but revert to a more normal phenotype when cultured at 37°C. Immorto-Bmpr2^R899X ^PMVEC were collected from adult mice as previously described[21], and verified by staining for endothelial markers VWF, PECAM, and VE-Cadherin.

### Affymetrix arrays

Mouse Gene 1.0 microarrays (Affymetrix, Foster City, CA) were performed on pooled RNA from three plates each. Array results were submitted to the NCBI gene expression and hybridization array data repository (GEO, http://www.ncbi.nlm.nih.gov/geo/), accession number pending. Arrays were analyzed using a combination of Affymetrix Expression Console, comparisons within Microsoft Excel, and Webgestalt for gene ontology groupings[22].

### Statistics

Statistical analysis was performed with Statview 5.0 (SAS Institute, Cary NC). Specific tests used are listed in figure legends, with p < .05 considered significant.

## Results

### Tie2-Bmpr2^delx4+ ^mice have increased RVSP, muscularization of vessels, and cellular proliferation

**Figure 1:**
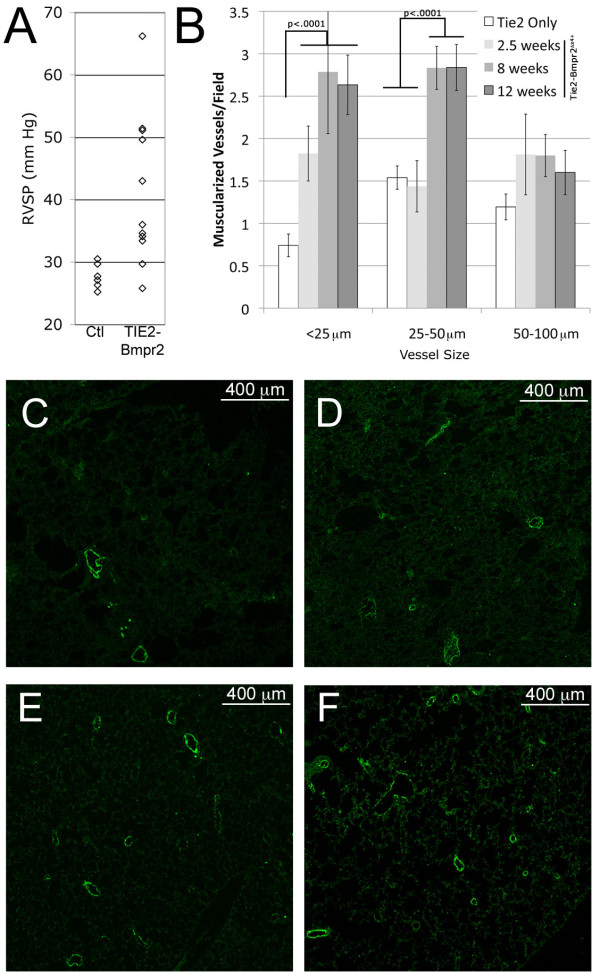
**RVSP and muscularization are increased in Tie2- Bmpr2^delx4+ ^mice**. **(A) **Scatter plot showing RV systolic pressures from 11 Tie2- Bmpr2^delx4+ ^mice and 6 control mice after 12 weeks' doxycycline. **(B) **Graph of muscularization of vessels visualized by actin staining showing a statistically significant increase in vessels under 25 μm compared to control at all time points, and an increase in muscularized vessels 25-50 μm compared to controls at the 8 and 12 week time points (p < .0001 by ANOVA with post-hoc t-test). There is never a statistically significant increase in muscularization in vessels over 50 μm. **(C-F) **Representative actin staining in control (C), 2.5 week (D), 8 week (E), and 12 week (F) time points, showing a progressive increase in small muscularized vessels.

Adult Tie2-rtTA x TetO_7_- Bmpr2^delx4+ ^(Tie2-Bmpr2^delx4+ ^for brevity) mice had the mutant Bmpr2 transgene activated in endothelium for 12 weeks through 1 g/kg doxycycline in chow; transactivator-only controls were similarly treated. Weight in both sexes was increased by about 20% (p < .01 by two-way ANOVA) in Tie2-Bmpr2^delx4+ ^mice compared to age- and sex- matched controls, as has been seen in our previous Bmpr2 models[9,14]. Tie2-Bmpr2^delx4+ ^mice also seemed to have a much higher frequency of fighting injuries, although we are not currently equipped to formally assess behavioral changes. Hemodynamic phenotype was evaluated by closed-chested cardiac catheterization, systemic pressure measurements by tail cuff, and muscularization of vessels was assessed by immunohistochemistry. Systemic pressures were normal (not shown), in the range of 90-120 mm Hg in both transactivator-only control and Tie2-Bmpr2^delx4+ ^mice. Closed-chested right ventricular systolic pressure (RVSP) was increased from 27 mmHg to 40 mmHg on average, and from 27 mm Hg to 35 mm Hg at the median (Figure 1A), significant at p = .003 by t-test with unequal variances, and p = .007 by Kruskal-Wallis (non-parametric) test.

Tissues were collected from additional groups of Tie2-Bmpr2^delx4+ ^with transgene activated 2.5 and 8 weeks to allow more complete analysis of histologic changes. There was an increase in muscularization of very small (<25 mm) vessels by 2.5 weeks, and an increase in both small and mid-sized vessels (25-50 mm) by 8 weeks; larger vessels never had a statistically significant increase in muscularization (Figure 1B-F).

**Figure 2:**
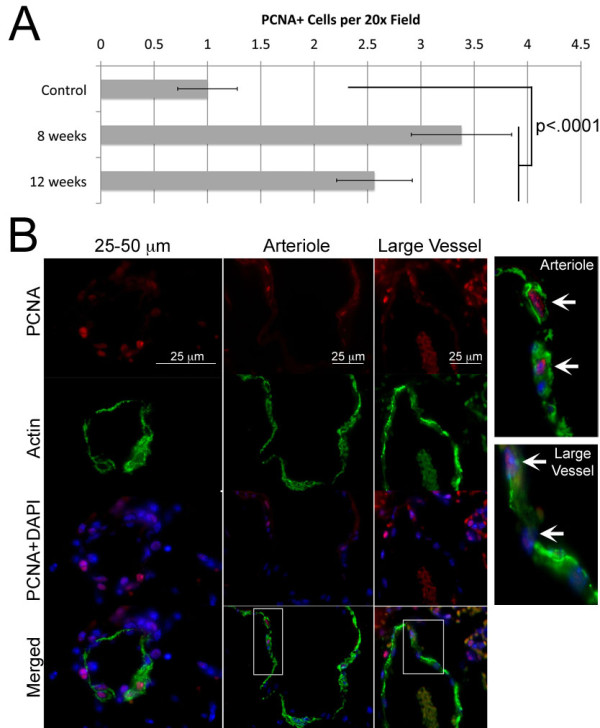
**(A) Quantification of nuclei with PCNA staining substantially greater than the median by histology indicates strong increases in proliferation in Tie2- Bmpr2^delx4+ ^mouse lung (p < .0001 by ANOVA with post-hoc t-test; no significant difference between 8 and 12 week time points) (B) Representative fields demonstrate that increased PCNA staining is found in vessels of all sizes**.

Tie2-Bmpr2^delx4+ ^mice showed about a threefold increase in proliferating cells in pulmonary vasculature, by counts of proliferating cell nuclear antigen (PCNA) positive cells (Figure 2). Increased PCNA staining was found in vessels of all sizes at 8 and 12 weeks.

### Tie2-Bmpr2^delx4+ ^mice have increased pulmonary inflammatory cells and thromboses

**Figure 3:**
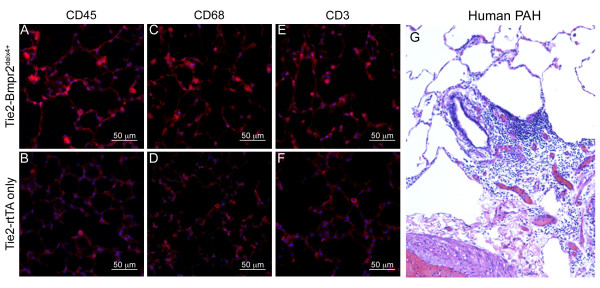
**Increased inflammation in Tie2-Bmpr2^delx4+ ^mouse lung compared to Tie2-rtTA only**. Blue is DAPI nuclear stain; red corresponds to pan-leukocyte marker CD45 (**A,B**), macrophage marker CD68 (**C,D**), or T-cell marker CD3 (**E,F**). In all cases, there are substantially more inflammatory cells in representative fields from Tie2-Bmpr2^delx4+ ^mouse lung than from control Tie2-rtTA only mouse lung. In contrast to this pattern, end-stage human lung is characterized by massive perivascular inflammation (**G**) which can be seen in Tie2-Bmpr2^delx4+ ^mice (Additional Figure 1), but is not typical. Human lungs were prepared with Carstairs' Stain.

Tie2-Bmpr2^delx4+ ^mice had a large increase in the number of inflammatory cells found throughout the lung, as assessed by immunofluorescence for the pan-leukocyte marker CD45 (Figure 3A,B). This included an increase in both CD68+ macrophages (Figure 3C,D) and CD3+ T-cells (Figure 3E,F). Aside from a small number of alveolar macrophages, inflammatory cells are rarely found in control mouse lungs. Human PAH is characterized by massive perivascular inflammation, even around relatively normal vessels (Figure 3G). While we did see occasional perivascular inflammation, primarily of CD3+ cells, it was not as common or dramatic as in human PAH (Additional File 1, **Figure S1**).

**Figure 4:**
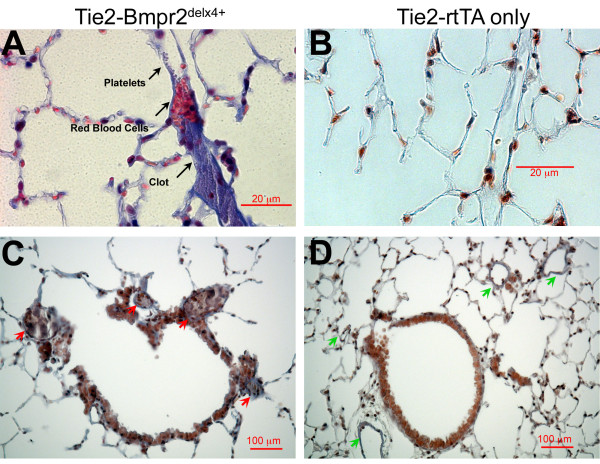
**Tie2-Bmpr2^delx4+ ^mouse lung has strongly increased frequency of thromboses in both small (A) and large (C) vessels**. We have never seen these in control lungs (**B **and **D **for comparison). Lungs were prepared with Carstairs' stain as described in the methods section

Tie2-Bmpr2^delx4+ ^mice also showed large numbers of thrombi in vessels (Figure 4). We do not believe that these could be an artifact of collection: our standard protocol for lung harvest includes pre-harvest perfusion with heparin and phosphate buffered saline, which generally clears the vessels; however, even when it does not, the result is vessels with loose collections of red blood cells, not a mature clot. Clots could be found in vessels of all sizes, from very small pre-capillary vessels (Figure 4A,B) to the large arteries adjacent to airways (Figure 4C,D).The frequency was variable from mouse to mouse and even across areas of lung in the same mouse, although there were areas in which most vessels were occluded (Additional File 2, **Figure S2A**). This inconsistency matches findings in human PAH, in which the same mutation can result in frequent thrombotic lesions in some patients, but not in others [23-25].

Thrombosis and perivascular inflammation could be found together (Additional File 2, **Figure S2B**), but this was uncommon.

### Loss of endothelial BMP signaling results in apoptosis in vivo and in culture

**Figure 5:**
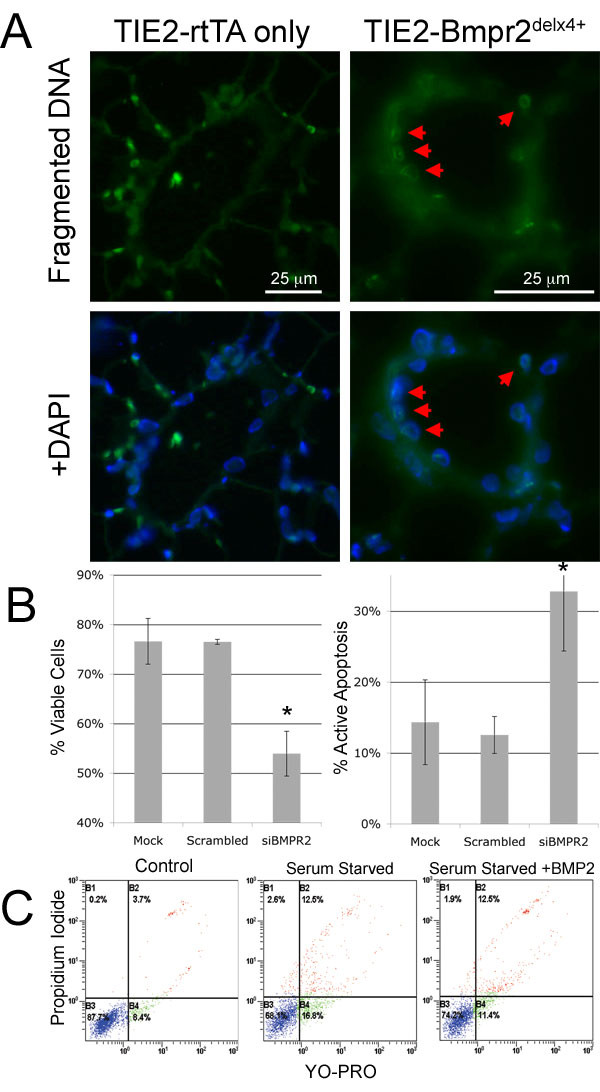
**Bmpr2^delx4+ ^mutation predisposes to increased PMVEC apoptosis**. (A) Immunofluorescence of fragmented DNA labeled with fluorescein; red arrows point to positive nuclei (B) PMVEC from control animals show increased apoptosis when inhibited with siRNA directed to BMPR2 * = p < .05 by ANOVA. (C) Representative scatter plots indicating exogenous BMP2 reduces apoptosis in serum starved condition.

Studies to analyze the effects of defective Bmpr2 on apoptosis *in vivo *and *in vitro *were conducted. *In vivo*, apoptotic cells were identified by detecting fragmented DNA in tissue obtained from mice treated for 12 weeks with doxycycline. Apoptosis is shown localized to the endothelium of vessels (Figure 5A). However, from histology, it was difficult to determine how widespread the problem was. *In vitro*, mouse cultured endothelial cells responded to inhibition of Bmpr2 by siRNA with substantially increased apoptosis (Figure 5B) as previously reported in human ECs[26]. In addition, BMP2 protects ECs from apoptosis under serum starved conditions (Figure 5C).

### Molecular consequences of Bmpr2^delx4+ ^and Bmpr2^R899X ^mutations in PMVEC

PMVEC were cultured from double and triple transgenic mice, expressing the Immortomouse transgene[20], the Rosa26-rtTA2 transgene[27], and either no additional transgenes (control PMVEC), the TetO_7_-Bmpr2^R899X^, or the TetO_7_-Bmpr2^delx4+ ^transgene. The immortomouse transgene was added because it is otherwise impractical to collect enough PMVEC from mice to perform experiments. Affymetrix Mouse Gene 1.0 microarrays were used to determine molecular consequences of Bmpr2^delx4+ ^and Bmpr2^R899X ^mutations in PMVEC. Two arrays were run for each cell type, with RNA for each array consisting of a pool of three independently grown plates (total of six arrays).

Using a requirement of a minimum of a 1.4x change, a minimum expression intensity of the higher of the pair of at least 128 (arbitrary Affymetrix units), and average expression values separated by more than the sum of their standard deviations (which equates to roughly a p < .05 for difference), we found 840 unique genes with gene ontology information differentially expressed in Bmpr2^delx4+ ^compared to controls (Additional File 3, Table S1), and 1692 differentially expressed in Bmpr2^R899X ^mutants compared to controls (Additional File 4, Table S2).

**Figure 6:**
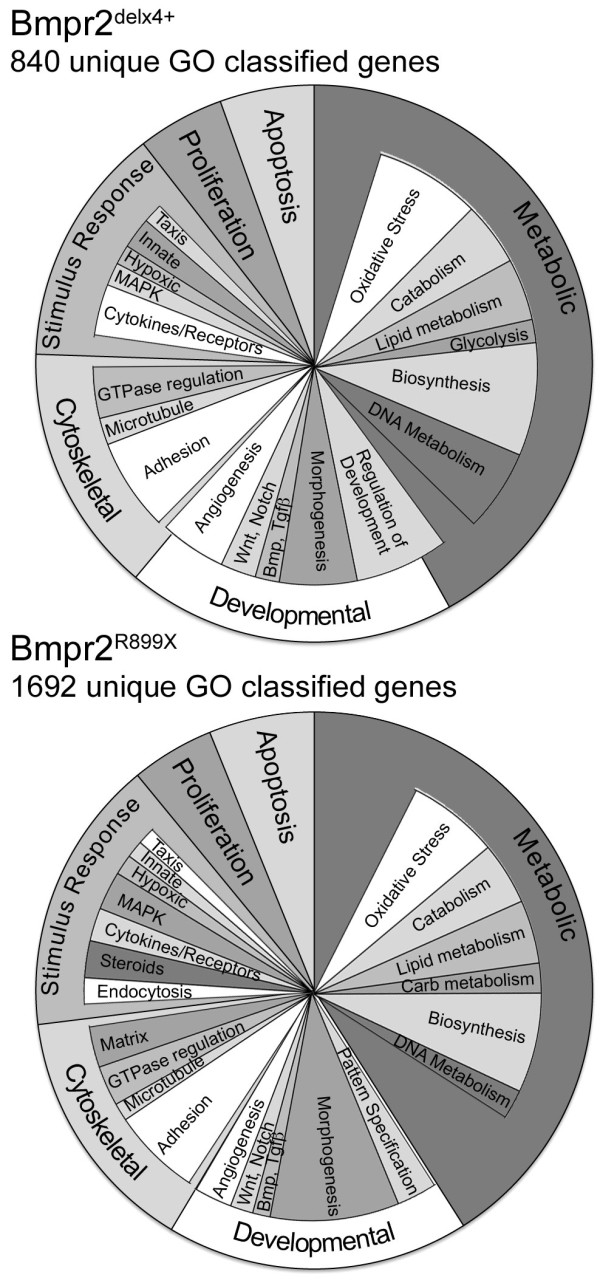
**Gene ontology classification of genes dysregulated in PMVEC cultured from BMPR2^delx4+ ^mice (top) or Bmpr2^R899X ^mice (bottom) indicates very similar patterns of gene dysregulation between mutation types**. Angular width of sections corresponds to proportion of genes within that ontology group. Outer labels correspond to top-level gene ontology groups: inner circle corresponds to more detailed gene ontology classification, all from the biological process tree. Most genes fall into more than one lower-level category, and so lower-level categories selected are meant to be representative, but are somewhat arbitrary. Full GO listing is included in Supplemental Tables 1 and 2.

Distribution of gene ontology groups in these genes is nearly identical between Bmpr2^delx4+ ^and Bmpr2^R899X ^PMVEC (Figure 6), consisting of metabolic, developmental, cytoskeletal, stimulus-response, proliferation, and apoptosis-related genes. This distribution of gene ontology groups is very similar to those seen as a common consequence of different categories of BMPR2 mutation in cultured vascular smooth muscle cells[27], with differences discussed below. In most ontology groups, direction of changes are also concordant between mutation types; the exceptions are inflammatory and thrombotic genes, as discussed below.

**Figure 7:**
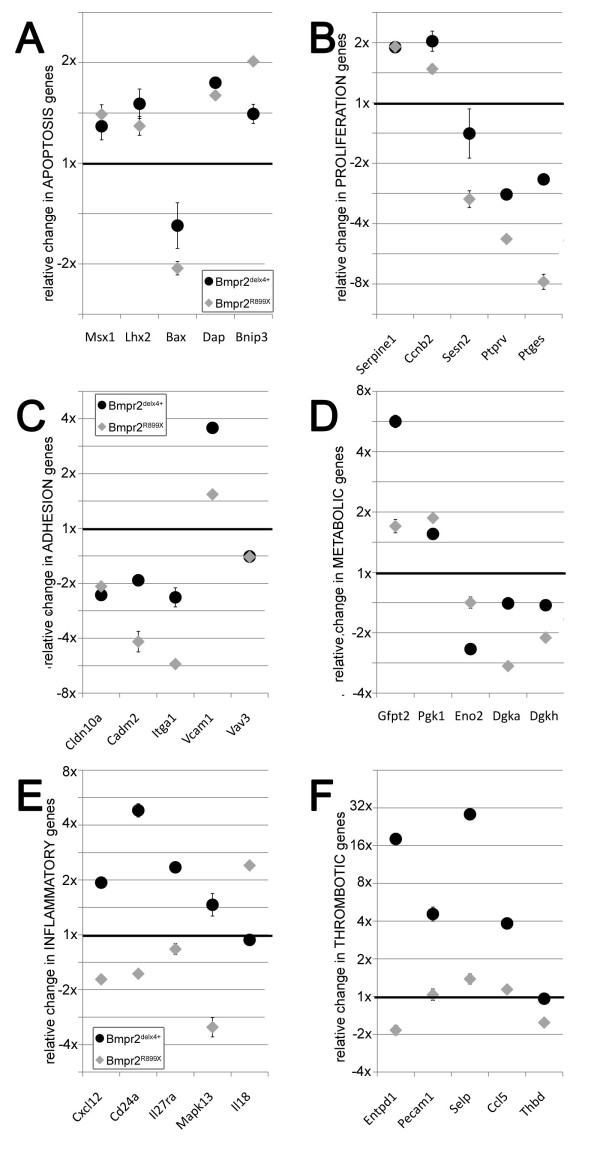
**Scatter plots indicating changes in illustrative genes from different gene ontology groups in Bmpr2^delx4+ ^(black circles) or Bmpr2^R899X ^(grey diamonds) PMVEC**. In each plot, the y-axis indicates fold change compared to control PMVEC on a log_2 _scale. Points falling on the 1x line are thus identical to controls; those above are upregulated, and those below are downregulated. Error bars indicate standard deviation in replicates; if they are invisible, they are smaller than the symbol (experimental replicates were very similar). Bmpr2^delx4+ ^and Bmpr2^R899X ^PMVEC had very similar changes in apoptosis(**A**), proliferation (**B**), adhesion (**C**), and metabolic (**D**) genes, but divergent changes in inflammatory (**E**) and thrombotic (**F**) genes.

We also found that within most specific pathways, the changes caused by expression of Bmpr2^delx4+ ^and Bmpr2^R899X ^in PMVEC were congruent. For apoptosis, we found common upregulation of Bmp target Msx1, known to drive apoptosis[28] (Figure 7A); overexpression of Msx1 is associated with capillary dropout[29]. Examples of other upregulated apoptosis-related genes include Lhx2, which complexes with Msx1[30], Death Associated Protein 1(Dap), and endothelial apoptosis gene Bnip3[31], while apoptosis inhibitor Bax is downregulated. We also found congruent alterations in multiple proliferation-related genes (Figure 7B). Examples include an increase in TGFbeta target SerpinE1 and in TGFbeta-mediated proliferation gene Cyclin B2(Ccnb2), and downregulation of Sestrin 2(Sesn2), key p53 target PTPRV[32], and prostaglandin e synthase (Ptges). Bmpr2^delx4+ ^and Bmpr2^R899X ^also showed matching alterations in adhesion related genes (Figure 7C), including for example downregulation of tight junction protein Claudin 10a (Cldn10a), intercellular adhesion molecule Cadm2, cell-cell adhesion molecule Itga1, and Vav3, a key regulator of Rho family GTPases especially as related to adhesion[33]. Finally, in metabolism (Figure 7D) we found concordant upregulation of oxidative stress-response genes such as Gfpt2[34] and glycolysis related genes such as Pgk1[35], with downregulation of gluconeogenesis gene Eno2[36], and diacylglycerol kinases Dgka and Dgkh, important in fatty acid metabolism and insulin resistance[37].

In contrast, expression of Bmpr2^delx4+ ^and Bmpr2^R899X ^mutations in PMVEC had very different effects on inflammatory and thrombotic genes. Bmpr2^delx4+ ^mutation resulted in induction of multiple cytokines not upregulated in Bmpr2^R899X ^(Figure 7E). There were many of these, but examples upregulated only in Bmpr2^delx4+ ^include the angiogenic chemokine Cxcl12[38], the interleukin 27 receptor Il27ra, and the inflammatory cell adhesion molecule CD24a. Some inflammatory genes were changed only in Bmpr2^R899X^, including downregulation of MAP kinase p38δ (Mapk13) and upregulation of the TH1 inducer interleukin 18 (Il18). We also tested expression of interleukin 6, which we had previously found to be regulated by Bmpr2^delx4+ ^mutation in smooth muscle; we found it upregulated in Bmpr2^delx4+ ^mutant PMVEC, but not in Bmpr2^R899X ^(Additional File 5, **Figure S3**).

Effects of the two mutations were also very different in thrombosis related genes (Figure 7F), with increases in prothrombotic genes in Bmpr2^delx4+ ^but not Bmpr2^R899X^; examples include 4x increases in platelet/endothelial cell adhesion molecule (PECAM1) and platelet chemokine ligand Ccl5, a 28x increase in platelet adhesion molecule selectin P (Selp), and an 18x increase in Entpd1, a nucleotidase involved in response to thrombosis[39]. In contrast, the anticoagulant thrombomodulin (Thbd) is downregulated, but only in Bmpr2^delx4+^.

It is also worth noting a set of changes we did not see. When we expressed Bmpr2 mutations in smooth muscle, either in vivo or in vitro, expression of the Bmpr2^delx4+ ^mutation resulted in a broad loss of smooth muscle markers[13,27]. It was clear from these data that maintenance of SMAD signaling was required for smooth muscle differentiation. Bmpr2 mutation did not cause a broad loss of endothelial markers in PMVEC. Table 1 shows a list of average expression of 14 endothelial markers; 7 are not substantially changed by either mutation, and only one, vascular cell adhesion molecule 1 (Vcam1) is consistently changed in both (it is upregulated in both).

**Table 1:** Endothelial markers are not consistently changed by Bmpr2 mutation.

	Control		Delx4		R899X		ANOVA	Post Hoc (p < .05)	Control
CD34	7135	0.5%	10325	5.3%	8001	0.1%	<.01	Delx4 > Control	7135
Endoglin	2191	7.5%	5572	2.9%	316	4.6%	<.0001	Delx4 > Control > R899X	2191
Endothelial-Specific Marker 1	103	4.8%	119	1.8%	122	0.3%	<.05	Delx4 = R899X > Control	103
Flk1 (VEGF receptor 2)	1928	0.8%	1711	10.3%	684	3.6%	<.01	Delx4 = Control > R899X	1928
Flt1 (VEGF receptor 1)	484	4.6%	312	5.4%	370	0.8%	<.01	Control > R899X > Delx4	484
Icam2	190	3.3%	276	0.7%	201	0.4%	<.001	Delx4 > Control = R899X	190
Multimerin 2	118	10.9%	127	0.4%	113	6.4%	NS		118
Pecam1	114	14.6%	520	10.8%	118	3.3%	<.01	Delx > Control = R899X	114
Selectin, endothelial cell	156	4.7%	167	10.2%	174	8.5%	NS		156
Thrombomodulin	4855	3.5%	4669	3.9%	3013	6.8%	<.01	Delx4 = Control > R899X	4855
Vcam1	2264	3.6%	8066	1.7%	3483	0.2%	<.0001	Delx4 > R899X > Control	2264
VE-Cadherin	172	5.4%	230	0.1%	144	13.2%	<.05	Delx4 > Control = R899X	172
Vegf-a	1308	4.1%	1176	6.1%	2283	11.7%	<.05	R899X > Control = Delx4	1308
Von Willebrand factor	206	12.1%	231	5.5%	232	1.0%	NS		206

Numbers are the average of two raw Affymetrix hybridization intensity numbers for each condition, with the standard deviation given as a percentage of that number. ANOVA was performed for significant differences between groups, followed by a post-hoc t-test for which groups were different.

## Discussion

This study analyzed the physiologic and molecular effects of Bmpr2 mutation in endothelium. We found that most Tie2-Bmpr2^delx4+ ^mice developed elevated RVSP, although with large variability in degree. This was associated with increased muscularization of small arteries (Figure 1), an increase in proliferating cells (Figure 2), inflammatory cells(Figure 3), thrombosis (Figure 4), and apoptosis (Figure 5). Molecular changes substantially matched the physiologic changes, with an increase in gene ontology groups related to proliferation (Figure 7B), apoptosis (Figure 7A), and thrombosis (Figure 7F). The increase in inflammatory cells seen is probably related both to the increase in cytokines (Figure 7E, Additional File 5) and the decrease in cell-cell adhesion genes (Figure 7C). BMPR2 has been previously shown to regulate endothelial barrier function in the context of leukocyte transmigration[40]. We also saw large scale alteration in metabolic genes, including response to oxidative stress (Figure 6); this matches our previous findings in smooth muscle and whole animals[27]. Unlike expression of Bmpr2^delx4+ ^mutation in smooth muscle, expression in PMVEC did not cause loss of differentiation markers. Overall, this is the first report of the molecular effects of Bmpr2 mutation in PMVEC, with results consistent with both our current physiology and our previous findings.

Physiologic results from this mouse were similar to results from an endothelial-specific BMPR2 knockout mouse described by Hong et al[41], despite having very different methods of generation. The only difference was higher penetrance in our model (~80% as opposed to ~30%). Histologic details such as thromboses and perivascular inflammatory cells and levels of increased proliferating cells were all almost identical between the models, which strongly increases confidence that both models accurately represent the consequences of complete suppression of signaling through BMPR2 in the endothelium.

Both of these models are quite different in histologic detail than expression of a different Bmpr2 mutation, Bmpr2^R899X^, in endothelium[15]. Endothelial expression of Bmpr2^R899X^, which retains intact Smad signaling[9], results in vascular pruning and increased RVSP, but without the obvious increase in thromboses. This also matches our molecular data; increases in thrombotic pathways were not seen in PMVEC with Bmpr2^R899X ^mutation (Figure 7F). Our molecular results imply that neither increased thrombosis nor increased inflammation are required for the development of Bmpr2-mediated PAH, although obviously both could exacerbate disease.

Broadly, we believe that the sum of the data to date suggests that the normal role of BMPR2 in the adult pulmonary vasculature is as a master switch mediating injury response. After injury, suppression of BMPR2 signaling through its cytoplasmic tail results in a decrease in cell-cell adhesion to improve migration and leukocyte recruitment, a metabolic shift towards glycolysis to support production of proteins and lipids needed for proliferation, and an increase in propensity for both proliferation and apoptosis to support tissue remodeling and angiogenesis or angeogenic repair. Suppression of BMPR2 signaling through SMAD results in an increase in thrombosis and an increase in expression of cytokines, and in smooth muscle but not endothelium, dedifferentiation. In those with intact BMPR2, this naturally resolves as the injury is repaired. In the case of BMPR2 mutation, these injury responses can never be successfully resolved, leading to a pathologic continuation of the pro-thrombotic and pro-proliferative pathways which would have been adaptive in acute injury.

Decades ago, long before molecular origins of PAH were known, there was controversy over whether the initiating events in the disease involved inflammation, proliferation, thrombosis, or vasoconstriction through defective endothelial-smooth muscle communication[42]. The discovery of BMPR2 as the familial PAH gene had promised a resolution to this controversy. However, the current study, as well as its predecessors in the literature, indicate that all of these are consequences of BMPR2 mutation alone, and our expression arrays suggest that they may all be relatively direct consequences of BMPR2 mutation, not an in vivo cascade. Our study does not address the issue of whether any are dispensable for the development of PAH. However, since each of these individually is capable of driving PAH in animal models[43], it is possible that inflammation, proliferation, thrombosis, and vasoconstriction in the context of pulmonary vascular injury can not be easily separated; it is not that one is the cause and the other are bystanders, but rather they are inextricably linked.

## Conclusions

We present here a relatively comprehensive first look at the physiologic and molecular consequences of Bmpr2 mutation. These data support an important role for Bmpr2 signal in maintaining endothelial homeostasis, and support our broad hypothesis that a normal role for Bmpr2 is in injury response. Future studies will be needed to determine the specific roles of and alterations in pathways identified, including, p53-mediated proliferation, MSX1-mediated apoptosis, a shift to glycolysis, and a loss of cell-cell adhesion.

## List of abbreviations

Bax: BCL2-associated X protein; Bnip3: BCL2/adenovirus E1B interacting protein 3; Cadm2: cell adhesion molecule 2; Ccl5: chemokine (C-C motif) ligand 5; Ccnb2: Cyclin B2; CD24a: CD24a antigen; Cldn10a: Claudin 10a; Cxcl12: chemokine (C-X-C motif) ligand 12; Dap: Death Associated Protein 1; Dgka: diacylglycerol kinase a; Dgkh: diacylglycerol kinase h; Eno2: enolase 2, gamma neuronal; Entpd1: ectonucleoside triphosphate diphosphohydrolase 1; Gfpt2: glutamine-fructose-6-phosphate transaminase 2; Il6: Interleukin 6; Il18: Interleukin 18; Il27ra: interleukin 27 receptor, alpha; IPAH: idiopathic pulmonary arterial hypertension; Itga1: integrin, alpha 1 [; Lhx2 - LIM homeobox protein 2; Mapk13 - mitogen activated protein kinase 13; Msx1: homeobox, msh-like 1; PAH: pulmonary arterial hypertension; PCNA: proliferating cell nuclear antigen; Pecam1: platelet/endothelial cell adhesion molecule 1; Pgk1: phosphoglycerate kinase 1; PMVEC: pulmonary microvascular endothelial cell; Ptges: prostaglandin e synthase; Ptprv: protein tyrosine phosphatase, receptor type, V; Selp: selectin P; SerpinE1: serpin peptidase inhibitor, clade E, member 1; Sesn2: Sestrin 2; TGFbeta: transforming growth factor, beta 1; Thbd: thrombomodulin; Vav3: vav 3 oncogene; Vcam1: vascular cell adhesion molecule 1.

## Competing interests

The authors declare that they have no competing interests.

## Authors' contributions

SM assisted in the animal experiments, analyzed the histology, and helped to draft the manuscript. MH conducted the siRNA experiments. TB created the murine PMVEC lines and conducted the affymetrix array experiments. JH performed the hemodynamic phenotyping and conducted immunohistochemistry. JJ assisted in development and testing of murine PMVEC lines. RG and HP provided the Tie2-rtTA mice and assisted in design of the experiments. DC assisted in model development and molecular studies. JL provided patient samples, assisted in study design, and helped draft the manuscript. EN performed and analyzed histology. KS participated in the design of the study. JW conceived of the study, participated in its design and coordination, and drafted the manuscript. All authors have read and approved the final manuscript.

## Supplementary Material

Additional file 1 **Figure S1**. Examples of perivascular inflammation in lungs from Tie2-Bmpr2^delx4+ ^mice with transgene activated 12 weeks. All images are from Tie2-Bmpr2^delx4+ ^mice. Blue = DAPI nuclear stain, green = actin, red = CD45 (left two images) or CD3 (right two images). Roughly 15% of vessels had more than 1-2 associated leukocytes. Click here for file

Additional file 2 **Figure S2**. **(A) **Some regions of some Tie2-Bmpr2^delx4+ ^lungs had occlusion of most vessels present (red arrowheads). In general, though, level of thrombosis was irregular from region to region within a lung, and of variable degree between individual mice. (B) Some thromboses (red arrowhead) had increased nearby inflammatory cells (white arrowheads, red = CD45 staining), but this was not typical. Click here for file

Additional file 3 **Table S1**. Genes differentially expressed in pulmonary microvascular endothelial cells expressing the Bmpr2^delx4+ ^mutationn as compared to controls. Click here for file

Additional file 4 **Table S2**. Genes differentially expressed in pulmonary microvascular endothelial cells expressing the Bmpr2^R899X ^mutation as compared to controls. Click here for file

Additional file 5 **Figure S3**. Interleukin 6 expression is increased in lungs from Bmpr2^delx4+ ^mice with transgene activated 12 weeks **(A) **and in PMVEC derived from Bmpr2^delx4+^, but not Bmpr2^R899X^, mice **(B) **by quantitative RT-PCR. Error bars are standard deviation; normalization is to HPRT as indicated on the vertical axis. For (A), each box is a separate animal, p < .05 by unpaired t-test. For (B), each box is a separate plate, p < .01 for delx4+ difference, p = NS for R899X difference by ANOVA with post-hoc t-test. Click here for file
