# Supplementary figures and images for: Physiologic and molecular consequences of endothelial Bmpr2 mutation
Source: Respir Res. 2011 Jun 22;12(1):84. doi: 10.1186/1465-9921-12-84 (PMC3141420; doi:10.1186/1465-9921-12-84)

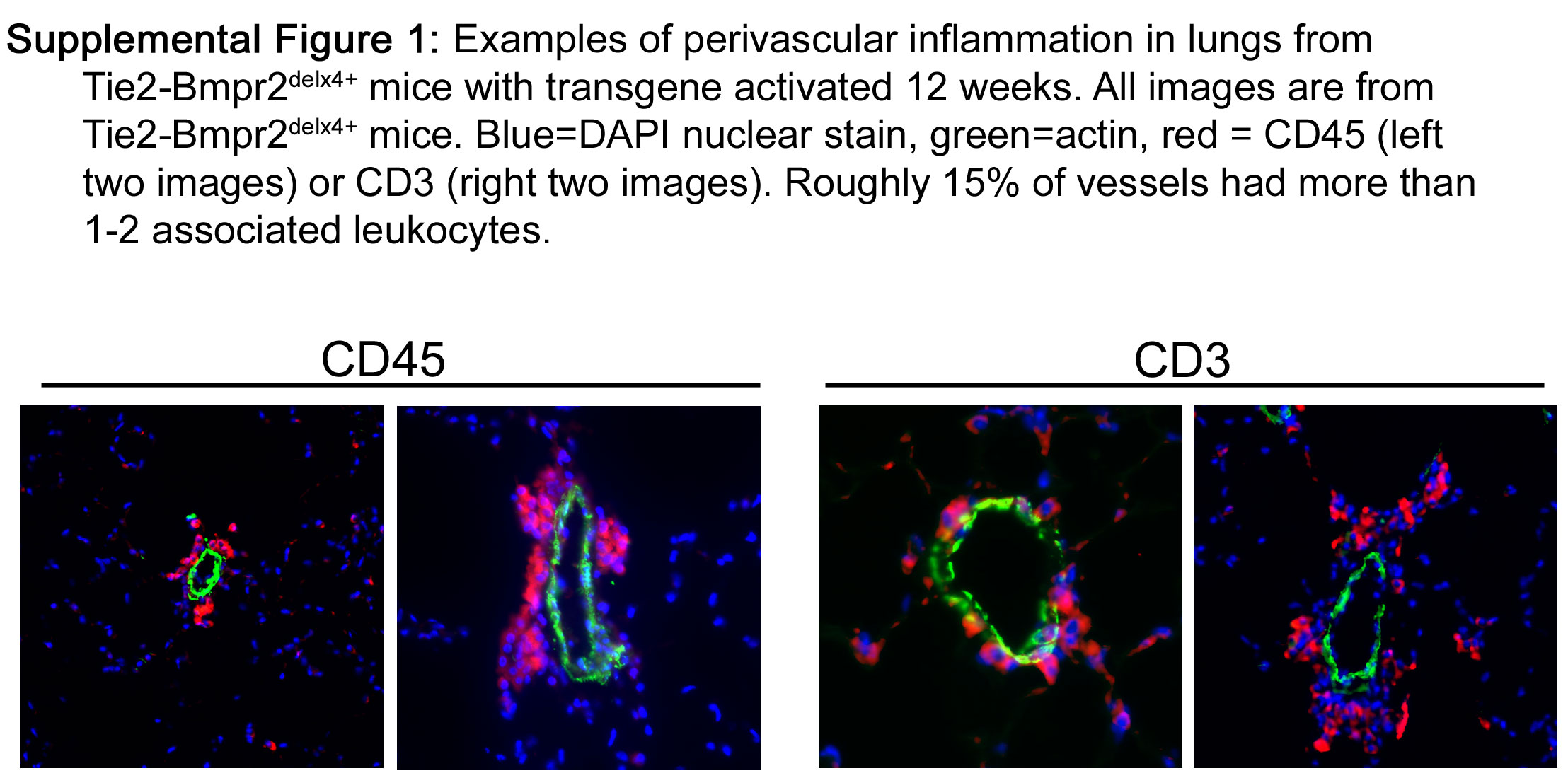

Supplement: Additional file 1 — Figure S1. Examples of perivascular inflammation in lungs from Tie2-Bmpr2delx4+ mice with transgene activated 12 weeks. All images are from Tie2-Bmpr2delx4+ mice. Blue = DAPI nuclear stain, green = actin, red = CD45 (left two images) or CD3 (right two images). Roughly 15% of vessels had more than 1-2 associated leukocytes. [file 1465-9921-12-84-S1.JPEG]

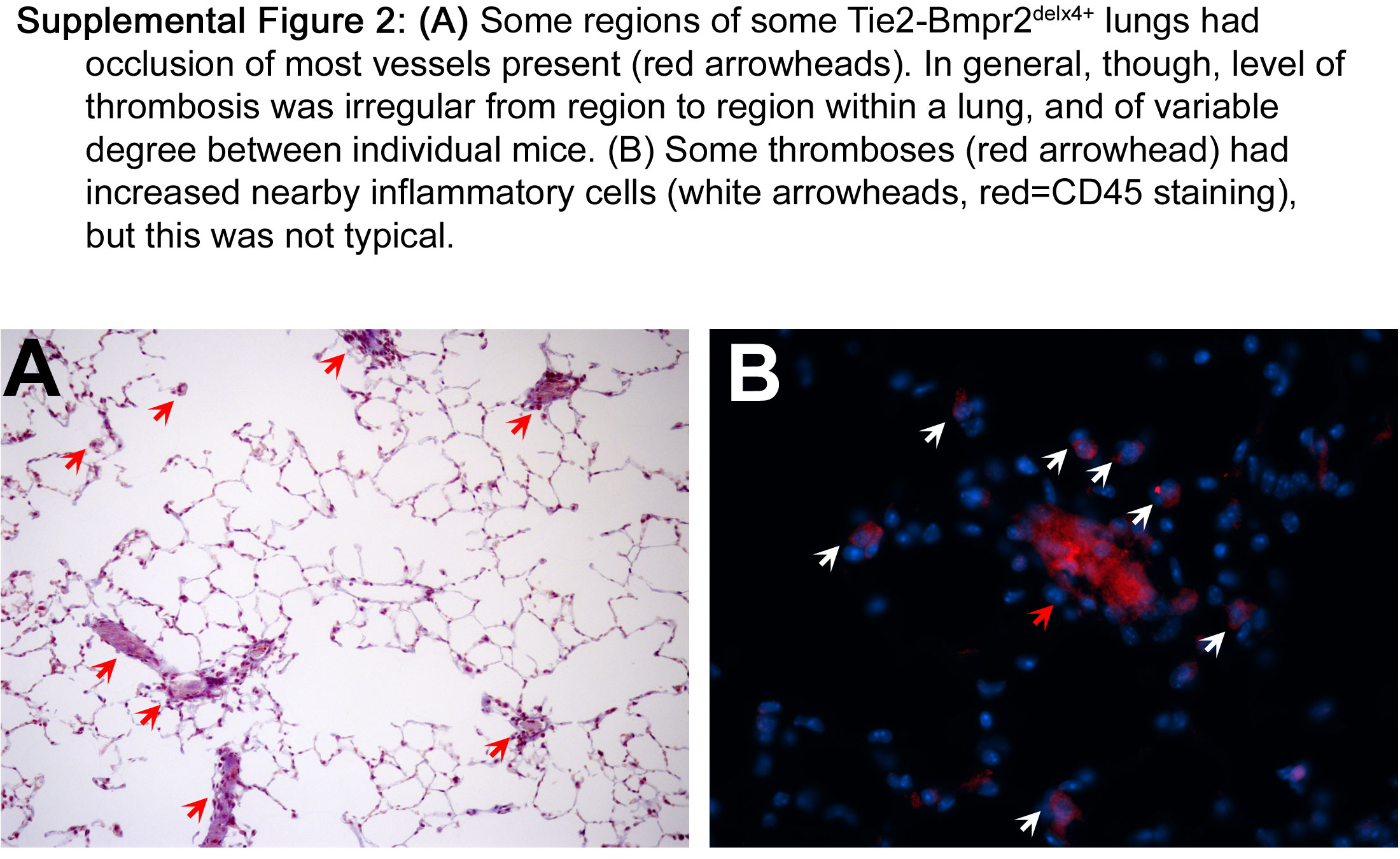

Supplement: Additional file 2 — Figure S2. (A) Some regions of some Tie2-Bmpr2delx4+ lungs had occlusion of most vessels present (red arrowheads). In general, though, level of thrombosis was irregular from region to region within a lung, and of variable degree between individual mice. (B) Some thromboses (red arrowhead) had increased nearby inflammatory cells (white arrowheads, red = CD45 staining), but this was not typical. [file 1465-9921-12-84-S2.JPEG]

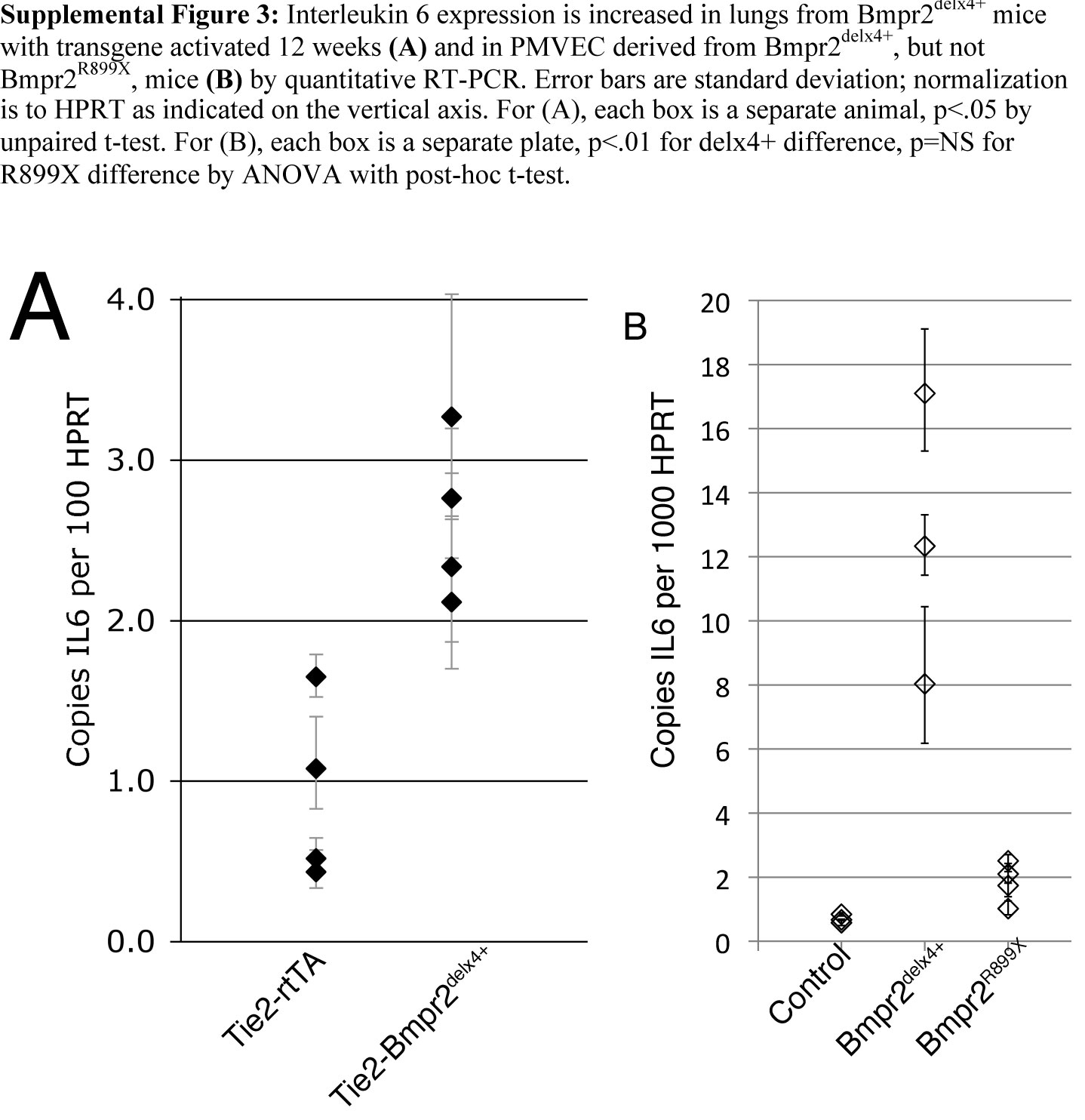

Supplement: Additional file 5 — Figure S3. Interleukin 6 expression is increased in lungs from Bmpr2delx4+ mice with transgene activated 12 weeks (A) and in PMVEC derived from Bmpr2delx4+, but not Bmpr2R899X, mice (B) by quantitative RT-PCR. Error bars are standard deviation; normalization is to HPRT as indicated on the vertical axis. For (A), each box is a separate animal, p < .05 by unpaired t-test. For (B), each box is a separate plate, p < .01 for delx4+ difference, p = NS for R899X difference by ANOVA with post-hoc t-test. [file 1465-9921-12-84-S5.JPEG]
